# Functional Analysis of Type III Effectors in *Xanthomonas campestris* pv. *campestris* Reveals Distinct Roles in Modulating *Arabidopsis* Innate Immunity

**DOI:** 10.3390/pathogens13060448

**Published:** 2024-05-24

**Authors:** Jing Huang, Hao Zhou, Min Zhou, Nana Li, Bole Jiang, Yongqiang He

**Affiliations:** 1 State Key Laboratory for Conservation and Utilization of Subtropical Agro-Bioresources, College of Life Science and Technology, Guangxi University, Nanning 530005, China; jhuang@gxu.edu.cn (J.H.); bolej@163.com (B.J.); 2 Guangxi Key Laboratory for Polysaccharide Materials and Modifications, Guangxi Minzu University, Nanning 530006, China

**Keywords:** *Xanthomonas campestris* pv. *campestris*, type III effector, plant immunity, patterns-triggered immunity, salicylic acid signaling pathway

## Abstract

*Xanthomonas campestris* pv. *campestris* (*Xcc*) is a significant phytopathogen causing black rot disease in crucifers. Its virulence relies heavily on the type III secretion system (T3SS), facilitating effector translocation into plant cells. The type III effectors (T3Es) disrupt cellular processes, promoting pathogen proliferation. However, only a few T3Es from *Xcc* have been thoroughly characterized. In this study, we further investigated two effectors using the T3Es-deficient mutant and the *Arabidopsis* protoplast system. XopE2*_Xcc_* triggers *Arabidopsis* immune responses via an unidentified activator of the salicylic acid (SA) signaling pathway, whereas XopL*_Xcc_* suppresses the expression of genes associated with patterns-triggered immunity (PTI) and the SA signaling pathway. These two effectors exert opposing effects on *Arabidopsis* immune responses. Additionally, we examined the relationship between the specific domains and functions of these two effector proteins. Our findings demonstrate that the N-myristoylation motif and N-terminal domain are essential for the subcellular localization and virulence of XopE2*_Xcc_* and XopL*_Xcc_*, respectively. These novel insights enhance our understanding of the pathogenic mechanisms of T3Es and contribute to developing effective strategies for controlling bacterial disease.

## 1. Introduction

The Gram-negative bacterial genus *Xanthomonas* comprises 27 species that collectively infect approximately 400 different host plants [1]. Most *Xanthomonas* species utilize the type III secretion system (T3SS) to directly translocate type III effectors (T3Es) into host cells, where they interfere with key steps in plant immune responses. After long-term coevolution with host plants, the known T3Es exhibit diverse structures and functions, disrupting the plant immune system in various ways, including inhibiting patterns-triggered immunity (PTI) or effector-triggered immunity (ETI), ultimately leading to plant infection [2,3,4]. Despite the identification of numerous T3Es, the virulence targets and molecular mechanisms remain incompletely understood.

*Xanthomonas campestris* pv. *campestris* (*Xcc*), the causative agent of black rot disease in cruciferous crops, represents an economically significant pathogen [5]. *Xcc* infects various cruciferous plants, including the model plant *Arabidopsis thaliana* [6]. Previous studies have highlighted the pathogenic mechanisms of *Xcc*, identifying over 100 genes contributing to its pathogenicity [7,8]. In the *Xcc* 8004 genome, 34 putative genes encoding T3Es [7] were identified. However, only several of them, including AvrXccC, XopAC, XopD, XopL, XopN, XopJ, and XopAM, have been functionally investigated [9,10,11,12,13,14,15,16,17].

XopE2*_Xcc_* acts as an Avr protein, resulting in the avirulence of *Xcc* 8004 on the resistant host plant Chinese cabbage cv. Zhongbai-83 [8,18]. A previous study revealed that XopE2*_Xcc_* was regulated by *hrpG* and *hrpX*, and the product was translocated into plant cells in a T3SS-dependent manner [17]. Similarly, XopL*_Xcc_*, a leucine-rich repeats protein encoded by the gene *XC_4273,* is crucial for *Xcc* 8004 pathogenicity. XopL*_Xcc_*, a virulence factor, suppresses PTI signaling in *Arabidopsis* [15]. However, the underlying mechanisms of these T3Es remain incompletely understood. Further investigations are necessary to understand the pathogenicity of XopE2*_Xcc_* and XopL*_Xcc_.* In this study, a T3Es-deficient mutant and a protoplast system were employed to investigate the function of the two effectors on the host plant *Arabidopsis thaliana*. These findings contribute to unveiling the molecular mechanisms of T3Es and aid in developing new strategies to control *Xcc* infections.

## 2. Material and Methods

### 2.1. Bacterial Strains and Growth Conditions

*Xcc* strains were cultured at 28 °C in either a nutrient broth-yeast extract (NYG) medium or minimal medium (MMX). Antibiotics were added as necessary at the following concentrations: rifampicin (50 μg/mL), ampicillin (50 μg/mL), and kanamycin (50 μg/mL for *E. coli* and 25 μg/mL for *Xcc*). For growth curve analysis, *Xcc* strains were pre-cultured in 5 mL of NYG until reaching OD_600_ = 1.0. Subsequently, for growth assessment in the MMX medium, pre-cultures were washed with MMX and inoculated in 300 μL of MMX at OD_600_ = 0.1. For growth assessment in the NYG medium, pre-cultures were inoculated in 300 μL of NYG at OD_600_ = 0.01. Optical density was measured every 12 h in MMX and every 4 h in NYG using an Automated Microbiology Growth Curve Analysis System.

### 2.2. Vector Constructions

Full-length DNA fragments of XopE2*_Xcc_* (*XC_2602*) and XopL*_Xcc_* (*XC_4273*) were amplified from *Xcc* 8004. For protoplast transient expression, PCR products were cloned into the pXSN-HA vector [19] to generate hemagglutinin (HA)-tagged constructs. For subcellular localization, PCR products were fused to the yellow fluorescent protein (YFP) in the pA7-YFP vector [20] under the control of the *Cauliflower mosaic virus* (*CaMV*) 35S promoter to generate C-terminal EYFP-tagged constructs. Site-directed mutagenesis was employed to introduce the myristoylation mutation G2A in XopE2*_Xcc_*. Plasmids were verified through DNA sequencing. Primer details are provided in Table S1.

### 2.3. Arabidopsis Protoplast Transient Expression

*Arabidopsis thaliana* Columbia (Col-0) was cultivated at 22 °C and 70% relative humidity. Protoplasts were prepared and transfected as previously described [21]. Briefly, leaves from 5- to 6-week-old plants were used for protoplast isolation. Enzyme solutions containing Cellulase R10 and Macerozyme R10 (Yakult, Tokyo, Japan) were used for leaf digestion. Plasmid DNAs were purified using the HiSpeed Plasmid Mini Kit (QIAGEN, Dusseldorf, Germany) in accordance with the manufacturer’s instructions. Subsequently, isolated protoplasts were transfected with 20 μg plasmid DNA using the polyethylene glycol (PEG)-calcium method (20 µg per 1 × 10^6^ cells). After a 6-h incubation period, protoplasts were exposed to the flg22 peptide (a 22-amino acid sequence from the N-terminal region of flagellin) for 1 h. Protoplasts transfected with an empty vector served as controls.

### 2.4. Gene Expression Analyses

Total RNA was isolated from protoplasts using Trizol Reagent (Solarbio, Beijing, China). First-strand cDNA was synthesized from 500 ng of total RNA using a PrimeScript RT reagent kit (Takara, Tokyo, Japan) in accordance with the manufacturer’s instructions. For real-time RT-qPCR, 20 ng of cDNA was mixed with SYBR Premix Ex Taq (Takara, Tokyo, Japan) and analyzed in triplicate using a LightCycler^®^ 480 Real-Time PCR System (Roche, Basel, Switzerland). Gene expression levels were normalized to the reference gene *Atactin2*. Primer sequences are listed in Table S1.

### 2.5. Virulence Assays

Virulence assays of *Xcc* strains on Col-0 were conducted using mesophyll infiltration as previously described [22]. Briefly, *Xcc* 8004 was cultured in NYG medium with appropriate antibiotics for 1 d. Bacterial cells were diluted to a concentration of 10^6^ CFU/mL in 10 mM MgCl_2_, and 10 μL of the mixture was injected into the leaves of 5- to 6-week-old *Arabidopsis* plants. Plant samples were maintained under humid conditions post-inoculation. Bacterial growth in planta was monitored by sampling leaf discs at different time points (days 0, 1, 3, 5, and 7 post-inoculation) and quantifying bacterial densities in log CFU/leaf.

### 2.6. Subcellular Localization

Subcellular localization of EYFP fusion proteins in plant cells was determined by tagging effector proteins to the N-terminus of the EYFP protein. *Arabidopsis* mesophyll protoplasts were transformed with purified plasmid DNA (20 µg per 2 × 10^5^ cells). After 16 h of dark incubation at 23 °C, protoplasts were observed using a laser scanning confocal microscope (TCS SP8, Leica, Solms, Germany). YFP fluorescence was excited at 488 nm and detected in the 525–550 nm range. Autofluorescence of the mesophyll coat was visualized by excitation at 514 nm and detected in the 650–704 nm range.

## 3. Results

### 3.1. Distinct Contributions of XopE2*_Xcc_* and XopL*_Xcc_* to Xcc 8004 Virulence on Arabidopsis

**Figure 1:**
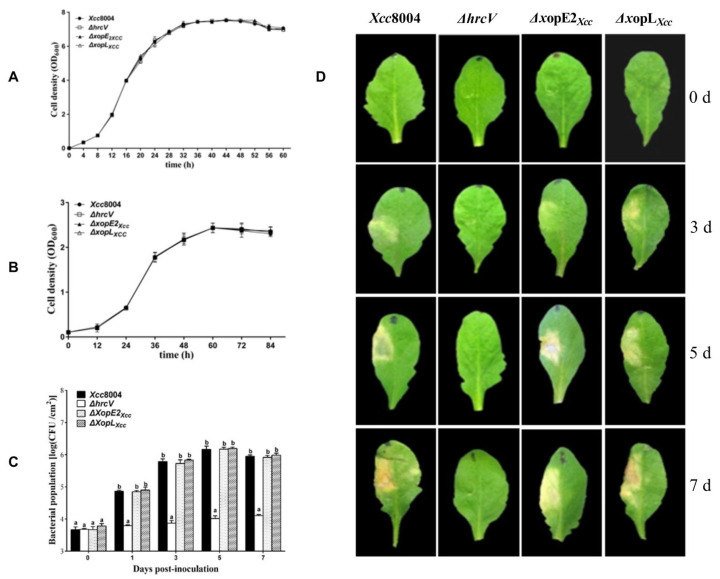
Pathogenicity analysis of *Xcc* 8004, Δ*xopE2_Xcc_*, and Δ*xopL_Xcc_* in *Arabidopsis*. (**A**) Grow curves in rich medium (NYG). (**B**) Grow curves in minimal medium (MMX). (**C**) Bacterial populations. (**D**) Disease symptoms. Δ*hrcV*, T3SS-defective mutant strain; Δ*xopE2_Xcc_*, XopE2*_Xcc_* mutant strain; Δ*xopL_Xcc_*, XopL*_Xcc_* mutant strain. The a\b labels shown on panel C represent significant differences (*n* = 30, *p* < 0.05, two-way ANOVA with Tukey’s HSD test). The same letters mean no statistical difference.

We investigated the impact of XopE2*_Xcc_* or XopL*_Xcc_* on *Xcc* 8004 pathogenicity in *Arabidopsis*. Growth curves analysis (Figure 1A,B) revealed similar cell densities among mutants Δ*xopE2_Xcc_*, Δ*xopL_Xcc_*, and *Xcc* 8004 in both NYG and MMX mediums. Moreover, no significant differences were observed in the bacterial population or disease symptom production among Δ*xopE2_Xcc_*, Δ*xopL_Xcc_*, and *Xcc* 8004 (Figure 1C,D). These results indicate that the deletion of XopE2*_Xcc_* or XopL*_Xcc_* does not significantly affect *Xcc* 8004 pathogenicity in *Arabidopsis*.

**Figure 2:**
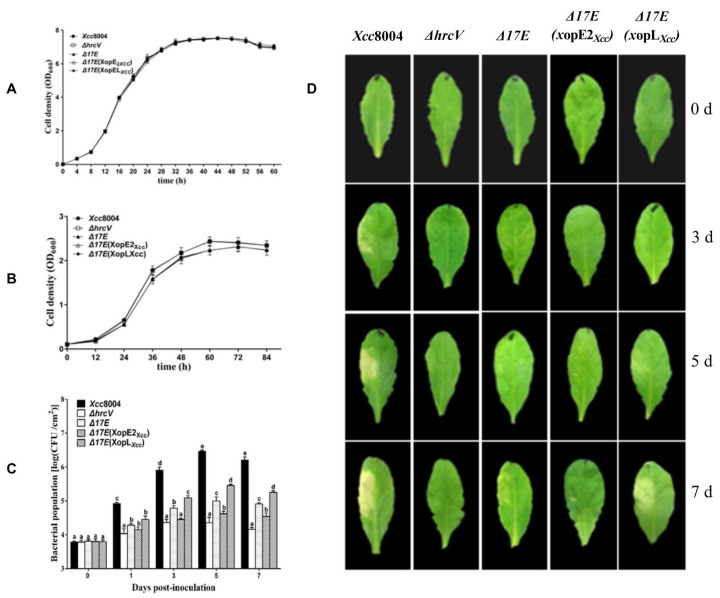
Pathogenicity analysis of *Xcc* 8004, Δ*17E, Δ17E*(*xopE2_Xcc_*), and Δ*17E*(*xopL_Xcc_*) in *Arabidopsis* under different conditions. (**A**) Grow curves in rich medium (NYG). (**B**) Grow curves in minimal medium (MMX). (**C**) Bacterial populations. (**D**) Disease symptoms. Δ*hrcV*, T3SS-defective mutant strain; Δ*17E*, 17 known T3Es-defficient mutant strain; Δ*17E*(*xopE2_Xcc_*), Δ*17E* strain containing XopE2*_Xcc_*; Δ*17E*(*xopL_Xcc_*), Δ*17E* strain containing XopL*_Xcc_.* The a\b\c\d\e labels shown on panel C represent significant differences (*n* = 30, *p* < 0.05, two-way ANOVA with Tukey’s HSD test). The same letters mean no statistical difference.

Furthermore, we generated a mutant lacking 17 known T3Es, including XopE2*_Xcc_* and XopL*_Xcc_*, designated as Δ*17E* (Table S2). Subsequently, XopE2*_Xcc_* and XopL*_Xcc_* were cloned into the pLAFR3 vector and introduced into the mutant Δ*17E* strain. The growth curves of Δ*17E*, Δ*17E*(*xopE2_Xcc_*), Δ*17E*(*xopL_Xcc_*), and *Xcc* 8004 were similar in both NYG and MMX mediums (Figure 2A,B). Following the same experimental procedure, these strains infiltrated into *Arabidopsis* leaves. Δ*17E* caused slight yellowing around the inoculation site, whereas Δ*17E*(*xopE2_Xcc_*) exhibited significantly milder disease symptoms and low bacterial proliferation, indicating that XopE2*_Xcc_* expression conferred resistance to *Xcc* 8004 infection in *Arabidopsis*. Conversely, the bacterial populations of Δ*17E*(*xopL_Xcc_*) were significantly higher than those of Δ*17E* and Δ*hrcV* in *Arabidopsis* plants but lower than those of *Xcc* 8004 (Figure 2C). Notably, Δ*17E*(*xopL_Xcc_*) induced severe disease symptoms, which were attributed to high bacterial titers (Figure 2D). These findings demonstrated the critical role of XopL*_Xcc_* in the virulence of *Xcc* 8004.

### 3.2. Effect of XopE2*_Xcc_* and XopL*_Xcc_* on Defense Resistance in Arabidopsis

**Figure 3:**
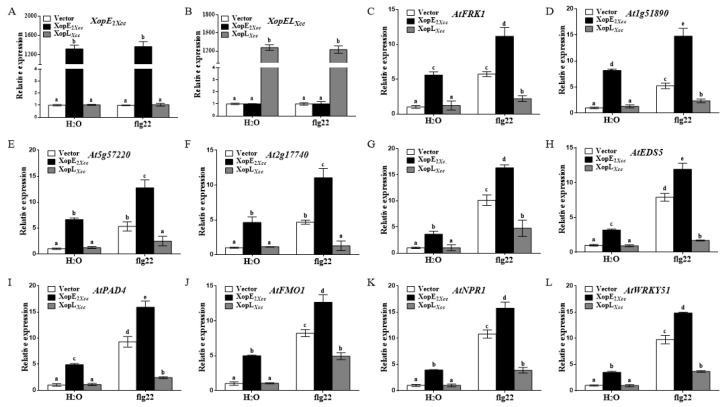
Influence of XopE2*_Xcc_* and XopL*_Xcc_* on the expression of defense-related genes in *Arabidopsis*. (**A**–**L**), The expression levels of defense-related genes in XopE2*_Xcc_* or XopL*_Xcc_*-transfected protoplasts of *Arabidopsis* Col-0 were measured using real-time qRT-PCR, respectively. The mRNA levels of all genes were normalized with *Atactin2*, and the relative expression levels were determined in protoplasts transfected with the control vector. The a\b\c\d\e labels represent significant differences (*n* = 5, *p* < 0.05, two-way ANOVA with Tukey’s HSD test). The same letters mean no statistical difference.

To investigate the impact of XopE2*_Xcc_* and XopL*_Xcc_* on defense resistance in *Arabidopsis*, we analyzed the expression of four PTI-related genes and six salicylic acid (SA)-related genes using RT-qPCR. The expression levels of the PTI-related genes were significantly higher in XopE2*_Xcc_*-expressing protoplasts than in the control group (Figure 3A–F). In contrast, the expression levels of these genes were significantly weaker in XopL*_Xcc_*-expressing protoplasts than those in the control. These results suggest that XopE2*_Xcc_* can activate the expression of PTI-related genes, whereas XopL*_Xcc_* possesses the ability to inhibit their expression in *Arabidopsis*.

All SA-related genes were significantly upregulated in XopE2*_Xcc_*-expressing protoplasts (Figure 3G–L). Notably, *AtSID2* expression was upregulated approximately 7.1-fold in control protoplasts, whereas it increased by approximately 18.3-fold in XopE2*_Xcc_*-expressing protoplasts. Conversely, XopL*_Xcc_* downregulated the expression of these genes. These findings indicate that XopE2*_Xcc_* activates plant defense response-associated genes, including those involved in PTI and the SA signaling pathway. However, XopL*_Xcc_* exerts the opposite effect, suggesting contrasting impacts on defense resistance in protoplasts.

**Figure 4:**
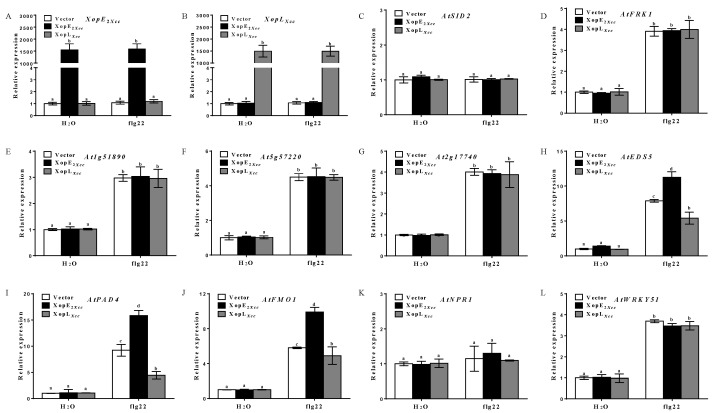
Influence of XopE2*_Xcc_* on the expression of defense response-associated genes in *sid2* mutant protoplasts. (**A**–**L**), The expression levels of defense-associated genes in XopE2*_Xcc_* or XopL*_Xcc_*-transfected protoplasts of *Arabidopsis sid2* mutant were measured using real-time qRT-PCR, respectively. The mRNA levels of all genes were normalized with *Atactin2*, and the relative expression levels were determined in protoplasts transfected with the control vector. The a\b\c\d labels represent significant differences (*n* = 5, *p* < 0.05, two-way ANOVA with Tukey’s HSD test). The same letters mean no statistical difference.

To further explore whether XopE2*_Xcc_* directly induces the SA signaling pathway, we analyzed the expression level of defense response-associated genes in *sid2* mutant protoplasts. All defense response-associated genes were induced in *sid2* mutant protoplasts expressing XopE2*_Xcc_*, albeit to a significantly lower extent than that in wild-type protoplasts (Figure 4), indicating that XopE2*_Xcc_* expression in SA-deficient plants does not enhance the expression of these defense response-associated genes. Notably, the expression levels of three genes (*AtEDS5*, *AtPAD4*, and *AtFMO1*) in the SA signaling pathway were significantly increased in *sid2* mutant protoplasts expressing XopE2*_Xcc_* (Figure 4H–J). These results suggest that XopE2*_Xcc_* boosts the expression of defense response-associated genes independently of SA accumulation, demonstrating a potential induction of upstream activators of the SA signaling pathway in *Arabidopsis*.

### 3.3. Subcellular Localization and Function Analysis of Mutant XopE2*_Xcc_* (G2A) Protein

**Figure 5:**
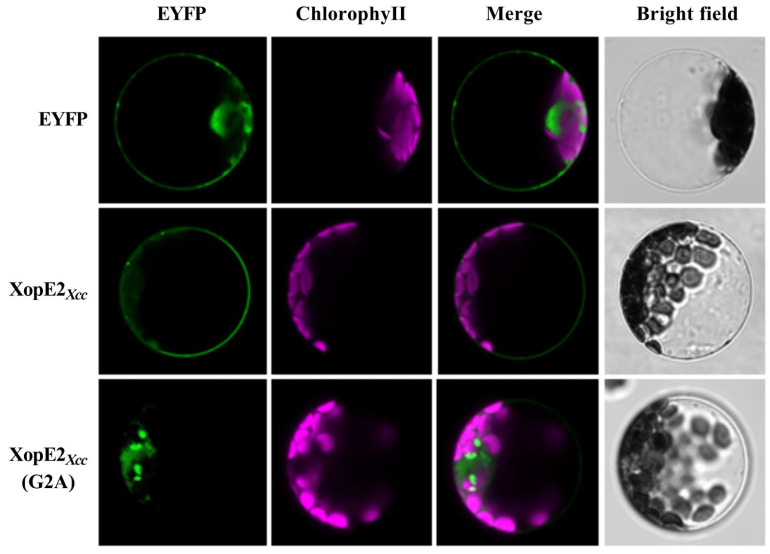
Subcellular localization of mutant XopE2*_Xcc_* (G2A) protein in protoplasts.

**Figure 6:**
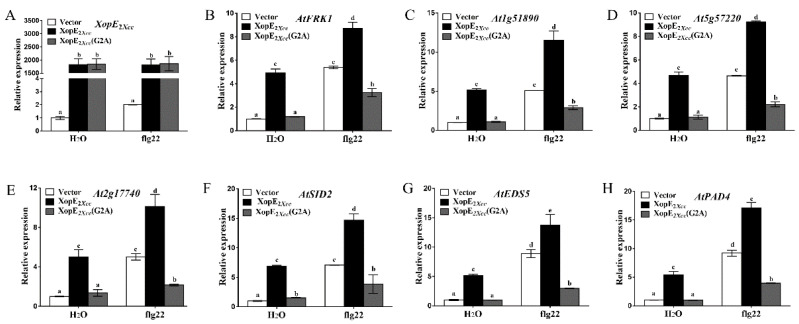
Influence of mutant XopE2*_Xcc_* (G2A) protein on the expression of defense response-associated genes in *Arabidopsis*. (**A**–**H**),The expression of response-associated genes in XopE2*_Xcc_* and XopE2*_Xcc_* (G2A) transfected protoplasts was measured using real-time qRT-PCR, respectively. The a\b\c\d\e labels represent significant differences (*n* = 5, *p* < 0.05, two-way ANOVA with Tukey’s HSD test). The same letters mean no statistical difference.

Myristoylation modification is crucial for anchoring proteins to cell membranes [23,24]. XopE members possess a conserved putative myristoylation motif (Met-Gly-X-X-X-Ser/Thr-) at the N-terminus. It was reported that the covalent attachment of myristate to the Gly residue is an important mechanism for protein anchor [3,23,25]. To assess the significance of the putative myristoylation site in XopE2*_Xcc_* for plasma membrane localization, we substituted the glycine (Gly2) residue in XopE2*_Xcc_* with Alanine (Ala), resulting in the mutant XopE2*_Xcc_* (G2A) protein fused with EYFP. These constructs were transiently expressed in *Arabidopsis* protoplasts. XopE2*_Xcc_* was localized to the plasma membrane in protoplasts. Conversely, XopE2*_Xcc_* (G2A) was detected in the cytoplasm surrounding chloroplasts and some undefined organelles, rather than the plasma membrane (Figure 5). Furthermore, the mutant XopE2*_Xcc_* (G2A) protein failed to induce the expression of defense response-associated genes (Figure 6). These findings indicate that the G2A mutation in XopE2*_Xcc_* disrupts its plasma membrane localization and its ability to induce defense responses, highlighting the dependence of XopE2*_Xcc_* function on myristoylation modification.

### 3.4. Subcellular Localization and Function Analysis of Various Mutant XopL*_Xcc_* Proteins

**Figure 7:**
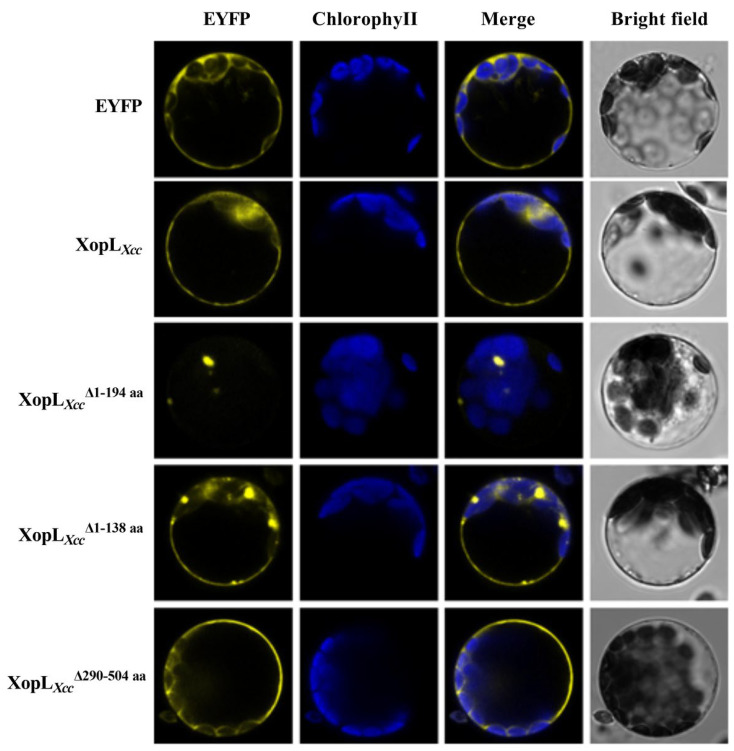
Subcellular localization of various mutant XopL*_Xcc_* proteins in protoplasts.

Full-length XopL*_Xcc_* cDNA and three mutants, XopL*_Xcc_*Δ^1-194aa^ (lacking the N-terminal domain [NTD] and Leucine-rich repeat [LRR1 and LRR2]), XopL*_Xcc_*Δ^1-138aa^ (lacking NTD and LRR1), and XopL*_Xcc_*Δ^290-504aa^ (lacking the C-terminal domain), were constructed. The results indicated strong EYFP fluorescence at the plasma membrane and in the cytoplasm in XopL*_Xcc_*-expressing protoplasts. The subcellular localization of XopL*_Xcc_*Δ^290-504aa^ was similar to that of XopL*_Xcc_*-expressing protoplasts, exhibiting membrane localization in protoplasts. XopL*_Xcc_*Δ^1-138aa^ showed EYFP fluorescence in some undefined organelles and the plasma membrane. Interestingly, XopL*_Xcc_*Δ^1-194aa^ exhibited subcellular localization surrounding chloroplasts, with no EYFP fluorescence observed in the protoplast membrane (Figure 7). These results demonstrate that the leucine-rich repeats (LRRs) of the N-terminal region of XopL*_Xcc_* play a critical role in protein localization in protoplasts.

**Figure 8:**
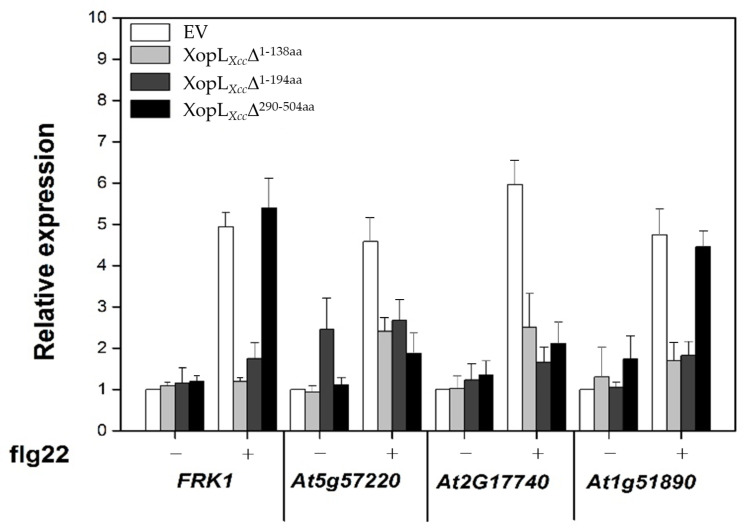
Influence of various mutant XopL*_Xcc_* proteins on the expression of defense response-associated genes in *Arabidopsis*.

Furthermore, considering the varied localization of the N-terminal truncated versions and wild-type XopL*_Xcc_* in *Arabidopsis* protoplasts, we evaluated the expression levels of defense-related genes. Notably, XopL*_Xcc_*Δ^1-138aa^ and XopL*_Xcc_*Δ^1-194aa^ significantly suppressed the expression of all four defense response-associated genes, while XopL*_Xcc_*Δ^290-504aa^ only negatively affected two marker genes, *At2g17740* and *At5g57220* (Figure 8). The N-terminal deletion of XopL*_Xcc_* altered the subcellular localization and function of this protein in plants, indicating that the N-terminal region of XopL*_Xcc_* may play a critical role in suppressing plant immunity.

## 4. Discussion

Plant pathogenic bacteria typically secrete T3Es into host cells to modulate host responses, facilitating successful infection and proliferation within the host plant. *Xcc* 8004 causes economically significant block rot diseases in various crops [26], underscoring the importance of elucidating the pathogenesis mechanisms of T3Es. However, the functions and host targets of many T3Es remain largely elusive. Demonstrating the virulence functions of numerous T3Es in *Arabidopsis*-*Xcc* 8004 model systems can be challenging due to functional redundancy or similar pathogenicity-related genes [27]. Our previous research highlighted the Avr function of XopE2*_Xcc_* in the host plant Chinese cabbage cv. Zhongbai-83 [8], whereas XopL*_Xcc_* was identified as critical for the full virulence of *Xcc* 8004 on the host plant Chinese radish [18]. Initial data from *Arabidopsis* infections did not reveal significant differences in disease symptoms or bacterial growth among Δ*xopE2_Xcc_*, Δ*xopL_Xcc_*, and the wild-type *Xcc* 8004 strain, possibly due to masking effects of other T3Es in *Xcc* 8004. To address potential redundancy issues, we developed a T3E-deficient mutant, the Δ*17E* strain, with 17 known T3E-coding genes deleted. Using this mutant strain, we demonstrated that XopL*_Xcc_* contributes to *Xcc* virulence, whereas XopE2*_Xcc_* functions as an Avr protein in *Arabidopsis*. These results confirm the presence of functional redundancy among certain effector proteins during infection of specific host plants. The Δ*17E* mutant strain serves as a valuable resource for the functional analysis of one or more T3Es.

### 4.1. XopE2*_Xcc_* Triggers Avirulence in Arabidopsis via Upstream Activation of the SA Signaling Pathway

The defense response-associated genes (*At1g51890*, *At2g17740*, *AtFRK1*, and *At5g57220*) are specifically induced by PTI from bacteria rather than other stress-related signals [28,29]. The expression levels of these genes were significantly elevated in *Arabidopsis* protoplasts transiently expressing XopE2*_Xcc_*, suggesting that XopE2*_Xcc_* activates their expression, thereby enhancing *Arabidopsis* PTI. Furthermore, SA-mediated immune responses play a critical role in *Arabidopsis* defense [4,30,31]. Our data revealed that expressing XopE2*_Xcc_* in *Arabidopsis* significantly activated the SA signaling pathway. Genes involved in SA biosynthesis (*AtSID2*), SA accumulation (*AtEDS5*, *AtPAD4*, and *AtFMO1*), and downstream signal transduction (*AtNPR1* and *AtWRKY51*) exhibited significantly higher expression levels in XopE2*_Xcc_*-transgenic plants than in control plants. Notably, XopE2*_Xcc_* expression in the SA defective mutant (*sid2* mutant) protoplasts failed to enhance the expression of four defense response-associated genes, confirming that XopE2*_Xcc_* elicits the *Arabidopsis* immune response possible through the SA signaling pathway. Moreover, the expression of genes involved in SA accumulation (*AtEDS5*, *AtPAD4*, and *AtFMO1*) in mutant protoplasts was significantly increased by XopE2*_Xcc_*, indicating that XopE2*_Xcc_* functions upstream of genes related to SA accumulation. These results suggest that XopE2*_Xcc_* triggers *Arabidopsis* immune responses via an unidentified activator upstream of the SA signaling pathway. Further identification and characterization of the target protein of XopE2*_Xcc_* in plants will enhance our understanding of how XopE2*_Xcc_* modulates plant defense responses.

N-myristoylation involves the covalent attachment of a hydrophobic 14-carbon saturated fatty acid to the glycine at residue two (Gly2), providing primary membrane targeting, signaling for several plant protein kinases and directing proteins to the plant cell plasma membrane [23]. Previous research has suggested that XopE2*_Xcc_*, a member of the XopE group, and possibly part of the transglutaminase superfamily, contains a putative N-myristoylation motif [3]. Consistent with findings on Avr*_Xcc_*C and XopE2*_Xe_*, our data indicate that the N-myristoylation motif is essential for the subcellular localization and avirulence function of XopE2*_Xcc_* in *Arabidopsis* [10,23].

Avr proteins are known to play a dual role in triggering ETI in plants with corresponding R proteins, while simultaneously suppressing plant defense in plants without the corresponding R proteins [32,33,34]. A previous study has demonstrated that XopE2*_Xcc_* (also known as avrXccE1) induces avirulence of *Xcc* 8004 in Chinese cabbage cv. Zhongbai-83, but not in *Xcc* strain 528^T^ on Early Jersey Wakefield cabbage [35]. Our data indicate that the avirulence function of XopE2*_Xcc_* in *Arabidopsis* is dependent on plasma membrane anchoring for host recognition, suggesting the presence of its corresponding R protein on the plasma membrane of *Arabidopsis*. Further identification and characterization of the R protein of XopE2*_Xcc_* in *Arabidopsis* are necessary to enhance our understanding of how XopE2*_Xcc_* modulates plant defense responses.

### 4.2. XopL*_Xcc_* Suppresses PTI-Related and SA-Related Genes in Arabidopsis

In this study, we observed significant suppression of four PTI-related genes in XopL*_Xcc_*-expressing protoplasts, suggesting its ability to inhibit *Arabidopsis* PTI. These findings are consistent with previous reports indicating that XopL*_Xcc_* enhances bacterial infection in *Arabidopsis* while suppressing the reactive oxygen species (ROS) burst, callose deposition, and other related responses [36]. Moreover, we observed significantly lower expression levels of SA-related genes in XopL*_Xcc_*-expressing protoplasts than in controls, suggesting that XopL*_Xcc_* promotes the synthesis and accumulation of SA, thereby suppressing *Arabidopsis* immune responses, possibly through the SA signaling pathway.

XopL*_Xcc_* belongs to the XopL superfamily, characterized by three LRR domains and a putative XopL E3 ligase box domain (XL box). XopL*_Xcv_* can suppress defense gene expression in plants and subvert plant immunity, with the XL box playing an essential role in E3 ubiquitin ligase activity and affecting plastid phenotypes [37,38]. However, our in vitro assessments did not reveal any E3 ligase activity for XopL*_Xcc_*. Conversely, a truncated form of XopL*_Xap_* (lacks the XL box) retained the ability to suppress plant immune responses [39]. Our research demonstrates that different N-terminal mutants exhibit varied localizations and effects on plant immunity, highlighting the essential role of the complete set of LRR domains of XopL*_Xcc_* in modulating plant immunity.

The LRRs are 20–29 residue sequence motifs present in numerous proteins with diverse functions. All proteins containing these repeats are believed to facilitate protein–protein interactions [40]. Various invasive bacterial proteins were identified to contain multiple LRR motifs [41]. The structure of XopL*_Xcc_* primarily consists of three LRRs, lacking known ligands and protein-anchoring sites in this region. The crystallographic study revealed that LRRs correspond to β-α structural units. These units are arranged so that the protein forms a curved structure [40]. It is hypothesized that the XopL*_Xcc_* protein shares a similar three-dimensional structure, and the LRRs are potentially responsible for protein anchoring and membrane localization. Truncation of the C-terminal region of XopL*_Xcc_* did not disturb protein conformation, as evidenced by its retained membrane localization ability. Further findings indicated that the membrane localization is likely mediated by the N-terminal region, possibly the LRRs, rather than the C-terminal part. We supposed that the parallel LRRs contribute to stabilizing the structure of β-sheets and α-helices through lateral interactions. Loss of the LRR region may lead to structural alterations that affect protein function.

## 5. Conclusions

Our study elucidates the divergent impacts of two T3E proteins on *Arabidopsis* immune responses. XopE2*_Xcc_* triggers *Arabidopsis* immune responses via an unidentified activator of the salicylic acid (SA) signaling pathway, whereas XopL*_Xcc_* suppresses the expression of PTI- and SA-related genes. The presence of the N-myristoylation motif and N-terminal domain proved crucial for the subcellular localization and virulence function of XopE2*_Xcc_* and XopL*_Xcc_*, respectively. These findings significantly enhance our understanding of the mechanisms employed by pathogenic bacteria T3Es and contribute to developing effective strategies for controlling bacterial diseases.

## Data Availability

Data presented in this study will be available from the corresponding author upon request.

## Supplementary Materials

The following supporting information can be downloaded at: https://www.mdpi.com/article/10.3390/pathogens13060448/s1, Table S1: Primers used in this work. Table S2: Bacterial strains and plasmids used in this work.
