# Supplementary material for: Functional Analysis of Type III Effectors in Xanthomonas campestris pv. campestris Reveals Distinct Roles in Modulating Arabidopsis Innate Immunity
Source: Pathogens. 2024 May 24;13(6):448. doi: 10.3390/pathogens13060448 (PMC11206781; doi:10.3390/pathogens13060448)
Supplement: Supplementary file 1 [file pathogens-13-00448-s001.zip › Table S1.pdf]

Table S1. Primers used in this work.

Purpose/ Name	Primer sequences (5'-3')
**Plasmid constructions**	
$xopE2_{Xcc}$-HA-F	TGTATGGATGGGTCTATGCGTTTCGAAGCCGA
$xopE2_{Xcc}$-HA-R	TGTATGGTCACCAACTCAAGGGCGGGC
$xopE2_{Xcc}$(G2A)-HA-F	TGTATGGGCTCTATGCGTTTCGAAGCCCCGA
$xopE2_{Xcc}$(G2A)-HA-R	TGTATGGTCACCAACTCAAGGGCGGGC
$xopL_{Xcc}$-HA-F	TGTATGGGTGTCAGCGGGCGGCCGGGT
$xopL_{Xcc}$-HA-R	TGTATGGCTAACTCTCAGAAGCGTCAG
$xopL_{Xcc}^{139}$-HA-F	TGTATGGGGGGAATTGCAAGAGCTCAC
$xopL_{Xcc}^{195}$-HA-F	TGTATGGAAAGATCTGACCCACCTAAA
$xopL_{Xcc}^{290}$-HA-F	TGTATGGCCTGACCATTGCACGATCCG
$xopE2_{Xcc}$-EYFP-F	CCGCTCGAGATGGGTCTATGCGTTTCGAAGCCGA
$xopE2_{Xcc}$-EYFP-R	GGACTAGTCCAACTCAAGGGCGGGCGAC
$xopE2_{Xcc}$(G2A)-EYFP-F	CCGCTCGAGATGGCTCTATGTTTCGAAGCCGA
$xopE2_{Xcc}$(G2A)-EYFP-R	GGACTAGTCCAACTCAAGGGCGGGCGAC
$xopL_{Xcc}$-EYFP-F	CCGCTCGAGGTGTCAGCGGGCGGCCGGGT
$xopL_{Xcc}$-EYFP-R	GGACTAGTACTCTCAGAAGCGTCAGTCG
$xopL_{Xcc}^{139}$-EYFP-F	CCGCTCGAGGGGGAATTGCAAGAGCTCAC
$xopL_{Xcc}^{139}$-EYFP-R	GGACTAGTACTCTCAGAAGCGTCAGTCG
$xopL_{Xcc}^{195}$-EYFP-F	CCGCTCGAGAAAGATCTGACCCACCTAAA
$xopL_{Xcc}^{195}$-EYFP-R	GGACTAGTCTAACTCTCAGAAGCGTCAG
$xopL_{Xcc}^{290}$-EYFP-F	CCGCTCGAGCCTGACCATTGCACGATCCG
$xopL_{Xcc}^{290}$-EYFP-R	GGACTAGTCTAACTCTCAGAAGCGTCAG
**Real-time PCR**	
AtFRK1-QF	ATCTTCGCTTGGAGCTTCTC
AtFRK1-QR	TGCAGCGCAAGGACTAGAG
At1g51890-QF	CCAGTTTGTTCTGTAATACTCAGG
At1g51890-QF	CTAGCCGACTTTGGGCTATC
At2g17740-QF	TGCTCCATCTCTCTTTGTGC
At2g17740-QF	ATGCGTTGCTGAAGAAGAGG
At5g57220-QF	AATGGAGAGAGCAACACAATG
At5g57220-QR	ATACTGAGCATGAGCCCTTTG
SID2-QF	TAAGGAGATAGAATCATTCGA
SID2-QR	GATGGGTCACTTCCAGCTACT
EDS5-QF	TCATATCCGAGATGCATAGAC
EDS5-QR	CATAGCCACTTCTTGTCACGA

PAD4-QF	TCTTCAGTTAAAGATCAAGGA
PAD4-QR	ATACAAAAGACGCGGAATGAC
FMO1-QF	GGAGATATTCAGTGGCATGCA
FMO1-QR	TTGATTAGCTAAAGCAGACTC
*NP*R*1*-QF	ATTGCCAAGGATTACGAAGT
*NP*R*1*-QR	TCTCACTCTGCTGCTGCTGTA
*W*R*KY51*-QF	GGAATCAACTAATAGAGGAAG
*W*R*KY51*-QR	TGTAATTACATAAGCTGCATC
$xopL_{Xcc}$-QF	AATCAACTCCTCGCCACTTAC
$xopL_{Xcc}$-QR	CGCAGATCAACCTCTTCCAG
$xopE2_{Xcc}$-QF	TTGACTCATGGTGCGATGG
$xopE2_{Xcc}$-QR	TCTGAATCCGTTTGTCCTGTC
Atactin2-QF	AGTGGTCGTACAACCGGTATTGT
Atactin2-QR	GAGGAAGAGCATTCCCCTCGTA

* The Sequences underlined indicate restriction site.
