# Supplementary material for: Functional Analysis of Type III Effectors in Xanthomonas campestris pv. campestris Reveals Distinct Roles in Modulating Arabidopsis Innate Immunity
Source: Pathogens. 2024 May 24;13(6):448. doi: 10.3390/pathogens13060448 (PMC11206781; doi:10.3390/pathogens13060448)
Supplement: Supplementary file 1 [file pathogens-13-00448-s001.zip › Table S2.pdf]

Table S2. Bacterial strains and plasmids used in this work.

Strains or plasmids	Relevant characteristic	Source
Xcc strains		
Xcc 8004	Wild type, Rif^r	Our lab's collection
Xcc 8004ΔhrcV	*hrcV* deletion mutant of 8004, Rif^r	Our lab's collection
Xcc 8004ΔXopE2$_{Xcc}$	XopE2$_{Xcc}$ deletion mutant of 8004, Rif^r	Our lab's collection
Xcc 8004ΔXopL$_{Xcc}$	XopL$_{Xcc}$ deletion mutant of 8004, Rif^r	Our lab's collection
Xcc 8004Δ17E	17 known Type III effectors (XC0052、XC0241、XC0541、XC0542、XC1210、XC1553、XC2004、XC2081、XC2210、XC2602、XC2994、XC2995、XC3160、XC3176、XC3177、XC3802 和 XC4273) deletion mutant of 8004, Rif^r	This work
Xcc 8004Δ17E(XopE2$_{Xcc}$)	*Xcc* 8004Δ17E harboring pLAFRJ-XopE2$_{Xcc}$, Rif^r, Tc^r	This work
Xcc 8004Δ17E(XopL$_{Xcc}$)	*Xcc* 8004Δ17E harboring pLAFRJ- XopL$_{Xcc}$, Rif^r, Tc^r	This work
Plasmids		
pLAFRJ	pLAFR3 derivative, containing pUC19 MCS, lacZ promoter, Tra-, Mob+, Tc^r	Our lab's collection
pLAFRJ-XopE2$_{Xcc}$	pLAFRJ containing the promoter and CDS of XopE2$_{Xcc}$, Tc^r	Our lab's collection
pLAFRJ-XopL$_{Xcc}$	pLAFRJ containing the promoter and CDS of XopL$_{Xcc}$, Tc^r	Our lab's collection
pUC19	Cloning vector, Amp^r	Our lab's collection
pA7-EYFP	Expression vector containing EYFP, Amp^r	Our lab's collection
pA7- XopE2$_{Xcc}$ -EYFP	pA7-EYFP containing XopE2$_{Xcc}$ -EYFP, Amp^r	This work
pA7 - XopE2$_{Xcc}$ (G2A)-EYFP	pA7-EYFP containing XopE2$_{Xcc}$ (G2A), in which the Gly2 residue in XopE2$_{Xcc}$ was replaced by Ala, Amp^r	This work

pA7 -XopL$_{Xcc}$-EYFP	pA7-EYFP containing XopL$_{Xcc}$-EYFP, Amp^r	This work
pA7-XopL$_{Xcc}^{139-504}$-EYFP	pA7-EYFP containing XopL$_{Xcc}^{139-504}$- EYFP, Amp^r	This work
pA7-XopL$_{Xcc}^{195-504}$-EYFP	pA7-EYFP containing XopL$_{Xcc}^{195-504}$-EYFP, Amp^r	This work
pA7-XopL$_{Xcc}^{290-504}$-EYFP	pA7-EYFP containing XopL$_{Xcc}^{290-504}$-EYFP, Amp^r	This work
pXSN-HA	Expression vector, Amp^r	(1)
pXSN-HA-XopE2$_{Xcc}$	pXSN-HA containing HA-XopE2$_{Xcc}$, Amp^r	This work
pXSN-HA-XopE2$_{Xcc}$ (G2A)	pXSN-HA containing HA-XopE2$_{Xcc}$ (G2A), Amp^r	This work
pXSN-HA-XopL$_{Xcc}$	pXSN-HA containing HA-XopL$_{Xcc}$, Amp^r	This work

1. Chen, S.; Songkumarn, P.; Liu, J.; Wang, G.L. A versatile zero background T-vector system for gene cloning and functional genomics. *Plant Physiology* **2009**, *150(3)*, 1111-1121.
